# Application of Indocyanine Green Angiography in Bilateral Axillo-Breast Approach Robotic Thyroidectomy for Papillary Thyroid Cancer

**DOI:** 10.3389/fendo.2022.916557

**Published:** 2022-06-23

**Authors:** Hui Ouyang, Baojia Wang, Botao Sun, Rong Cong, Fada Xia, Xinying Li

**Affiliations:** ^1^Department of Thyroid Surgery, Xiangya Hospital, Central South University, Changsha, China; ^2^National Clinical Research Center for Geriatric Disorders, Xiangya Hospital, Central South University, Changsha, China; ^3^Department of the Operating Room, Xiangya Hospital, Central South University, Changsha, China

**Keywords:** indocyanine green angiography, fluorescence, robotic thyroidectomy, papillary thyroid cancer, parathyroid grand

## Abstract

**Background:**

Indocyanine green angiography (ICGA) has been used to identify and preserve the parathyroid glands (PGs), and to evaluate PGs viability and function during thyroid surgery. However, evidence on the utilization of IGCA in thyroid cancer and robotic surgery is lacking. The efficacy of IGCA remains to be evaluated in PTC patients undergoing bilateral axillo-breast approach robotic thyroidectomy (BABA RT) and central neck dissection (CND).

**Methods:**

From March 2020 to August 2021, 81 papillary thyroid cancer (PTC) patients receiving total thyroidectomy and CND were enrolled in this retrospective analysis. An intravenous bolus of 7.5 mg ICG was administrated three times in the ICGA group (n=34). Medical records were reviewed and analyzed, including the baseline characteristics, surgical parameters, PGs-related parameters, and perioperative PTH and calcium levels.

**Results:**

The mean number of total identified PGs and preserved PGs were significantly more in the ICG group than in the control group (3.74 ± 0.45 vs. 3.15 ± 0.55, P<0.001; 3.12 ± 0.64 vs. 2.74 ± 0.57, P=0.007, respectively), as were PTH and calcium levels on POD 1 (23.16 ± 18.32 vs. 6.06 ± 7.74, P=0.039; 2.13 ± 0.11 vs. 2.08 ± 0.08, P=0.024, respectively). While there were no differences in PTH levels on POD 30. Additionally, patients with at least one well vascularized PG had higher ioPTH 3 and PTH on POD 1, which significantly suggested the absence of postoperative hypocalcemia. Although not statistically significant, ICGA seemed superior to relative ioPTH decline and ioPTH 3 in predicting postoperative hypocalcemia.

**Conclusion:**

In PTC patients undergoing BABA RT and CND, ICGA is a simple, safe, effective, and cost-effective tool in better identification and preservation of PGs as well as evaluation of PGs viability and function, with the potential to preserve more PGs, guide more appropriate autotransplantation, and accurately predict postoperative hypocalcemia.

## Introduction

The incidence of thyroid cancer has been increasing dramatically in recent decades, and the majority (>90%) of these newly diagnosed thyroid cancers are papillary thyroid cancer (PTC) (1). Surgical removal of the thyroid and the central compartment remains the mainstay of therapy. Recently, robotic thyroidectomy has become an accepted and preferred surgical option for selected PTC patients with comparable surgical outcomes and superior cosmetic outcomes (2). Hypocalcemia resulting from postoperative hypoparathyroidism is the most common complication associated with severe morbidity, reduced quality of life, and increased risk of mortality (3–5). The incidence of temporary and permanent hypocalcemia following thyroidectomy ranges from 19% to 38% and 0% to 3%, respectively (6). Postoperative hypocalcemia occurs mainly due to the accidental resection or devascularization of parathyroid glands (PGs). Thus, the preservation of the PGs and their vascularization are crucial for reducing the risk of postoperative hypocalcemia during thyroid surgery (3, 5, 6).

Over the years, the mainstays of parathyroid protection have been visual detection of PGs, capsular dissection technique, and autotransplantation of nonperfused glands (3, 5, 6). Recently, near-infrared fluorescence imaging (autofluorescence and indocyanine green angiography(ICGA)) has emerged as a novel intraoperative tool to help detect, identify, and preserve the PGs during thyroid surgery (7). Near-infrared autofluorescence is based on the endogenous fluorophore in PGs and facilitates the identification and preservation of PGs, thereby reducing the rate of postoperative hypoparathyroidism (7). However, the adoption of autofluorescence has certain drawbacks (8): Thyroid tissue might present similar autofluorescence intensities with the parathyroid tissue in some cases; False positives signals may also occur from colloid nodules, brown fat, and metastatic lymph nodes; most importantly, autofluorescence is unable to assess the perfusion status and vitality of PGs.

While ICGA allows the real-time assessment and direct imaging of tissue perfusion and vascularization. ICGA was initially used in ophthalmology to detect macular degeneration. Subsequently, its utility has expanded across multiple surgical procedures, such as intestinal anastomoses, cholangiography, and lymph node mapping (9). ICGA has been primarily used to assess perfusion and ongoing function of PGs after thyroidectomy (10–16), and many studies (10, 14, 15, 17, 18) have found a good correlation between ICG fluorescence of PGs and parathyroid function. On the other hand, ICGA could also be employed to identify and preserve PGs before the dissection, despite the fact that few articles have addressed this topic (16, 19). Furthermore, ICGA has lately gained attention for its ability to predict postoperative hypocalcemia in thyroid surgery (10, 14, 20). However, there are limited data regarding the utility of ICGA in thyroid cancer surgery (12, 21), especially when accompanied by central neck dissection (CND), and in the robotic system (21, 22). Thus, we conducted this study in PTC patients undergoing bilateral axillo-breast approach robotic thyroidectomy (BABA RT) and CND, to evaluate the potential role of ICGA for identification and preservation of PGs, evaluation of PGs viability and function, and prediction of postoperative hypocalcemia.

## Materials and Methods

### Patients

**Figure 1:**
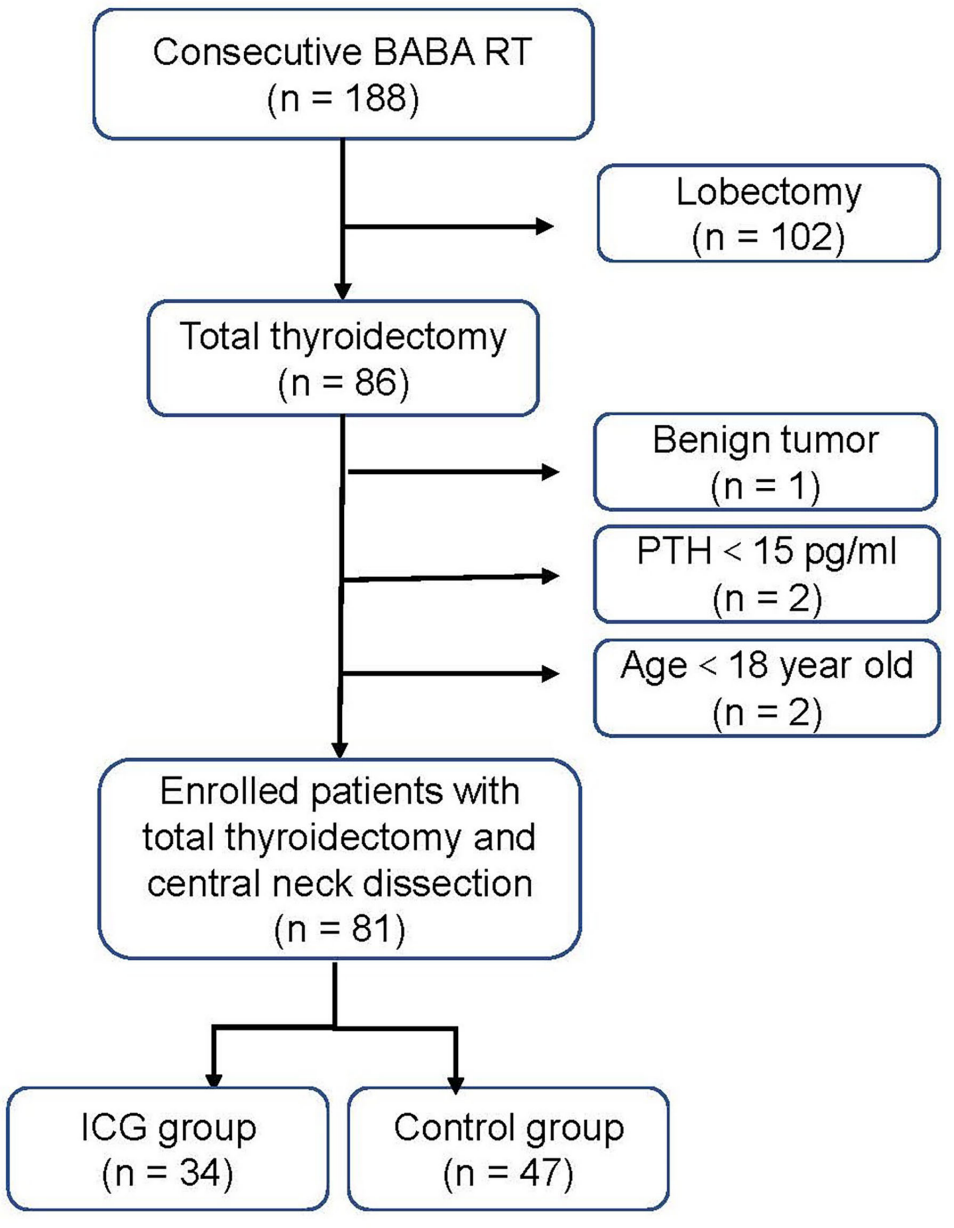
Flow diagram of the study patients. *BABA RT* bilateral axillo-breast approach robotic thyroidectomy. *PTH* parathyroid hormone.

From July 2020 to August 2021, 81 consecutive adult patients with PTC treated with BABA RT at the Department of Thyroid Surgery, Xiangya Hospital, were enrolled retrospectively. All patients underwent total thyroidectomy with unilateral or bilateral CND by a single experienced surgeon (Xinying Li). The patients were divided into two groups: the ICG group (34 patients) and the control group (47 patients) based on the use of ICGA. Patients with cosmetic requirements, 18 years and older, and postoperative pathologically confirmed PTC were included. Patients were excluded in case of allergy or intolerance to ICG or iodine dyes, severe renal or hepatic impairment, concurrent parathyroid disease or preoperative PTH ≤15 pg/mL, and previous thyroid or parathyroid surgery. A flowchart for the selection of patients is shown in **Figure 1**. All patients were followed at least six months after surgery. The present study complied with the Declaration of Helsinki and was approved by the Ethics Committee of Xiangya Hospital Central South University (No. 202108136). Written informed consents were obtained from all patients prior to inclusion.

### ICG

ICG (10 ml: 25 mg, Weicai Pharmaceutical Co Ltd, China.) is a water-soluble, albumin-bound, and FDA-approved fluorescent dye (23). Its fluorescent properties are in the near-infrared light wavelengths (peak absorption at 800 nm, peak emission at 830 nm), which penetrate the tissue up to 1 cm in-depth, allowing the surgeon to see behind the tissue surface (24). Thus ICGA enables good visualization of PGs and their vascularization in the thyroid surgical field. Additionally, the parathyroid fluorescence intensity depends on the amount of ICG taken up by PGs, capable of evaluating the perfusion and viability of PGs (14). ICGA can be repeatedly used because ICG has a half-life of between 2 to 3 minutes and is eliminated from the liver 15−20 minutes after injection, and the toxic dose is up to 5 mg/kg (15, 25).

### Surgical Management

The surgical techniques in BABA RT have been previously described in detail (26). All surgeries were performed on the DaVinci Xi^®^ systems (Intuitive Surgical, Sunnyvale, California, United States), equipped with a fluorescence imaging vision system(Firefly Fluorescence Imaging) to provide real-time endoscopic visible and ICGA imaging (NIR illuminator: 805 nm; filter: 825 nm). Video recordings on surgical procedures were made. All patients underwent total thyroidectomy with routine prophylactic or therapeutic CND. Bilateral CND was performed in case of bilateral tumor or isthmus tumor. We have routinely inspected the ex vivo of the specimen before sending it for histological examination to check out the possible presence of PGs. Notably, an immediate PGs autotransplantation was performed if the PGs could not be retained *in situ* due to devascularization or accidental removal. The parathyroid were minced into 1 mm fragments and injected into the ipsilateral deltoid muscle with a 1 ml syringe.

### ICGA and Intraoperative PTH Testing

**Figure 2:**
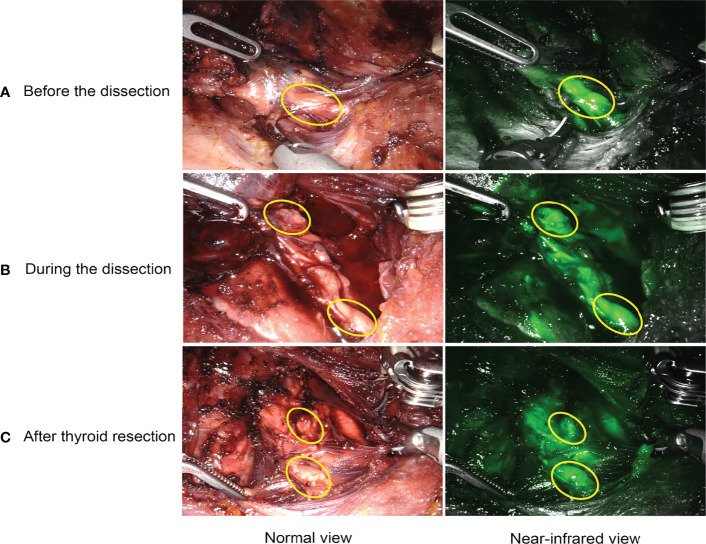
Intraoperative application of ICGA. **(A)** detection and identification of PGs. **(B)** real-time visualization and preservation of PGs and their vascularization during the dissection. **(C)** evaluation of viability and function of PGs after thyroid resection.

**Figure 3:**
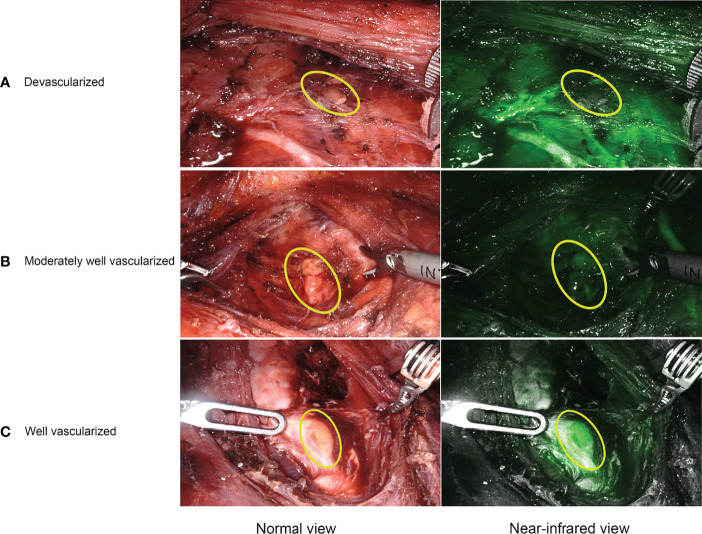
Representative ICGA images. **(A)** a devascularized parathyroid gland (ICG score 0). **(B)** a moderately well vascularized parathyroid gland (ICG score 1). **(C)** a well vascularized parathyroid gland (ICG score 2).

For ICGA imaging, 25 mg ICG powder was dissolved in 10 mL of saline, and 7.5 mg (3 mL) was administered intravenously at three different time points. The special procedures for IGCA were as follows: P1: after lateral mobilization of thyroid lobe and adequate exposure of ipsilateral central neck compartment, the first bolus was used to detect and identify PGs before the dissection (**Figure 2A**), as well as visualize and preserve PGs and their vascularization during the dissection (**Figure 2B**). P2: with the same aim as P1, the second bolus was given after removing one lobe and adequate exposure of the other lobe and central neck compartment. P3: the third bolus was performed after removing both the lobe and central neck compartment to evaluate the viability of the identified PGs in P1 and P2 (**Figure 2C**). The degree of parathyroid fluorescence was classified as 0, black (devascularized, **Figure 3A**); 1, gray/heterogeneous (moderately well vascularized, **Figure 3B**); and 2, white (well vascularized, **Figure 3C**), in line with previous studies (10–13, 15, 20, 25, 27).

Intraoperative measurement of parathyroid hormone (ioPTH) from a peripheral blood sample (4 mL) had been performed in the ICG group by a nurse after the induction of anesthesia, 10 minutes after removal of the first lobe, and 10 minutes after removal of both the lobe and central neck compartment, which were labeled as ioPTH 1, ioPTH 2, and ioPTH 3, respectively.

### Data Collection and Postoperative Management

The data included the general demographic and clinical characteristics; surgical parameters (Operation time and intraoperative blood loss); number of identified PGs, Preserved PGs in situ, autotransplanted PGs and Inadvertently resected PGs; PTH levels before the operation, during the intraoperation (ioPTH 1, ioPTH 2, ioPTH 3), and on postoperative day 1 and 30 (POD 1 and POD 30); Relative ioPTH decline ([ioPTH 1−ioPTH 3]/ioPTH 1); Serum calcium levels before the operation and on POD 1; ICG score; Postoperative hospital stay time. Transient postoperative hypoparathyroidism is defined as PTH on POD 1 lower than 15 pg/mL, and permanent hypoparathyroidism is defined as PTH level lower than 15 pg/mL at 6 months of follow-up. While temporary and permanent hypocalcemia are defined as a serum calcium level (< 2.0 mmol/L) at the same time points as the measured PTH levels. The inadvertently resected PGs are defined as those identified in the pathological report after surgery.

All patients received empirical intravenous calcium gluconate (0-2.0 g/day) as well as oral calcium (1.2-3.6 g/day) and calcitriol (0.25-0.75 μg/day) after surgery until discharge. The levels of PTH and calcium were measured on the first day and the first month after thyroid surgery to adjust doses of calcium and calcitriol. PTH is measured using an electrochemiluminescence immunoassay (Roche Elecsys System, Roche Diagnostics, Mannheim, Germany, reference values 15-65 pg/mL), and serum calcium is measured by the o-cresolphthalein complexone method (reference values 2.0-2.6 mmol/L).

### Statistical Analysis

Continuous variables were presented as mean ± standard deviation (SD) and compared using independent samples t-tests. Chi-square or Fisher exact tests were performed to analyze categorical data as appropriate.

To analyze the association of the use of ICGA with surgery outcomes (PTH on POD1, PTH on POD 30, calcium on POD 1, hypoparathyroidism, and hypocalcemia, identified PGs, preserved PGs *in situ* and autotransplanted PGs), univariate analyses were performed to estimate the crude coefficients or odds ratios, and their 95% confidence intervals (CIs). While multiple multivariable models were used to determine the adjusted coefficients or odds ratios, and their 95% CIs, after adjusting for potential confounders (age, gender, BMI, Hashimoto’s thyroiditis, extrathyroid extension, scope of CND). Due to the fact that repeated measurements of PTH levels and serum calcium were longitudinal data and highly correlated with each other, the generalized estimating equation model was used for intergroup comparison of PTH or calcium at each time point. Similarly, The linear regression model was also constructed as a sensitivity analysis to determine the relationship between the use of ICGA and postoperative PTH or calcium, with preoperative PTH or calcium as an additional confounder in the model. Additionally, the Propensity score-adjusted model was also built to assess the robustness of the results and determine the correlation between ICGA and binary dependent variables (hypoparathyroidism, hypocalcemia, identified PGs, preserved PGs in situ, and autotransplanted PGs). Propensity scores for the use of IGGA for each patient were built using the predicted probabilities resulting from a multivariable logistic regression model, with ICGA as the outcome and all potential confounders (as described above) as variables in the model. Then regression model was used to determine the effect of ICGA with the propensity score as a covariate.

The optimal cut-off values of ioPTH 3, relative ioPTH decline and PTH on POD 1 for predicting transient hypocalcemia were determined by the maximum value of the Youden index. The predictive ability of different predictors of postoperative hypocalcemia was assessed and compared using area under the Receiver Operating Characteristic curve (AUC). Diagnostic characteristics (sensitivity, specificity, positive and negative predictive values, and accuracy) of different early predictors (ioPTH 3, relative ioPTH decline, ICGA (ICG score 2)) were also assessed and compared.

The regression method was used to impute the missing values, including preoperative PTH levels (3/81, 3.7%), PTH on POD 1 (1/81, 1.2%), and PTH on POD 30 (12/81, 14.8%). All statistical analyses were conducted using R statistical software (version 4.1.1) and SPSS 26.0, and a two-tailed p-value < 0.05 was considered statistically significant for all the tests.

## Result

### Patient Characteristics

A total of 81 consecutive PTC patients receiving BABA RT were enrolled and analyzed (**Figure 1**). All patients underwent total thyroidectomy and CND without conversion to open surgery. The follow-up time ranged from 6 to 20 months. The baseline characteristics of the patients were summarized in **Table 1**. Among the 81 enrolled patients, 34 were in the ICG group (28 women (82.4%); mean (range) age, 34.41 ± 8.61 (18–52) years) and 47 were in the control group (37 women (78.7%); mean (range) age, 35.47 ± 9.40 (18–58) years). All baseline characteristics between the two groups were clinically similar. The missing values for preoperative PTH (3/81, 3.7%), PTH on POD 1 (1/81, 1.2%), and PTH on POD 30 (12/81, 14.8%) were imputed.

**Table 1:** Demographic and clinical characteristics of the patients in the two groups.

Variables	ICG Group(n = 34)	Control Group(n = 47)	P-value
Sex (male/female)	6/28	10/37	0.686
Age (years)	34.41 ± 8.61	35.47 ± 9.40	0.607
BMI (kg/m^2^)	22.62 ± 3.19	23.02 ± 3.01	0.565
Tumor size (mm)	12.12 ± 6.5	12.00 ± 7.23	0.930
Scope of CND (n)			0.750
Unilateral	22	32	
Bilateral	12	15	
Hashimoto’s thyroiditis (n)			0.639
No	22	28	
Yes	12	19	
Extrathyroid extension (n)			0.666
No	59	44	
Yes	5	6	
Preoperative PTH (pg/ml)	40.17 ± 13.29	37.53 ± 9.75	0.306
Preoperative calcium (mmol/L)	2.28 ± 0.10	2.30 ± 0.10	0.321

BMI, body mass index; CND, central neck dissection.

### Parathyroid Glands Identification and Preservation

**Figure 4:**
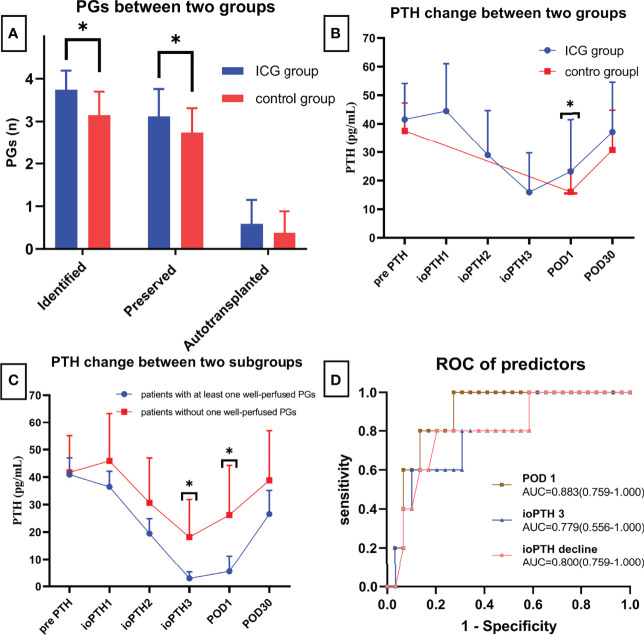
**(A)** Comparison of identified, preserved and autotransplanted PGs between two groups; **(B)** PTH changes between two groups; **(C)** PTH changes between patients with at least one well vascularized PG and patients without; **(D)** ROC of different predictors for postoperative hypocalcemia. * means statistically significant.

As shown in **Figure 4A** and **Table 2**, The mean number of total identified PGs was significantly greater in the ICG group than in the control group (3.74 ± 0.45 vs. 3.15 ± 0.55, P<0.001), as was the mean number of preserved PGs *in situ* (3.12 ± 0.64 vs. 2.74 ± 0.57, P=0.007). The mean number of autotransplanted PGs was slightly higher in the ICG group than in the control group, but not statistically significant (0.59 ± 0.56 vs. 0.38 ± 0.50, P=0.094). In addition, both groups had one inadvertently resected PGs. Moreover, the rate of patients with 4 identified PGs was higher in the ICG group than in the control group (25/37 vs. 11/47, P<0.001), so was the rate of patients with 4 PGs preserved *in situ* (9/34 vs. 3/47, P=0.021). Furthermore, after adjustment for confounders using a propensity score-adjusted model (**Table 3**), the use of ICGA was associated with a significantly higher rate of patients with 4 PGs identified and 4 PGs preserved *in situ* than nonuse (adjusted odds ratio, 9.02 [95% CI, 3.25-25.09], P<0.001; adjusted odds ratio, 5.12 [95% CI, 1.23-21.26], P=0.025) (**Table 3**). While the rate of patients with autotransplanted PGs was not significantly associated with the use of ICGA (adjusted odds ratio, 2.29 [95% CI, 0.91-5.80], P=0.079).

**Table 2:** Surgical outcomes of the patients in the two groups.

Variables	ICG Group (n = 34)	Control Group (n = 47)	P-value
Identified PGs	3.74 ± 0.45	3.15 ± 0.55	< 0.001*
Preserved PGs in situ	3.12 ± 0.64	2.74 ± 0.57	0.007*
Autotransplanted PGs	0.59 ± 0.56	0.38 ± 0.50	0.083
Identified PGs			< 0.001*
2	0	4	
3	9	32	
4	25	11	
Preserved PGs in situ			0.021*
2	5	15	
3	20	29	
4	9	3	
Autotransplanted PGs			0.117
Number = 0	15	29	
Number ≥ 1	19	18	
Inadvertently resected PGs			1.000
No	1	1	
Yes	33	46	
PTH on POD 1 (pg/ml)	23.16 ± 18.32	16.06 ± 7.74	0.039*
PTH on POD 30 (pg/ml)	36.97 ± 17.49	30.71 ± 13.90	0.077
Calcium on POD 1 (mmol/L)	2.13 ± 0.11	2.08 ± 0.08	0.024*
Transient hypoparathyroidism			0.764
No	27	36	
Yes	7	11	
Transient Hypocalcemia			0.779
No	29	39	
Yes	5	8	
Operation time (min)	88.82 ± 4.78	81.28 ± 8.81	< 0.001*
Intraoperative blood loss (ml)	12.94 ± 3.51	13.30 ± 4.46	0.688
Postoperative hospital stay time (days)	2.59 ± 0.61	2.94 ± 0.83	0.964
Transient hoarseness			0.961
No	31	43	
Yes	3	4	

PGs, parathyroid glands; POD, postoperative day. *Statistically significant.

**Table 3:** Coefficients or odd ratios of the use of ICGA vs. nonuse in diferent models with different surgery outcomes as response variables.

Response Variables	Univariate Analysis		Multiple Multivariate Models					
			Generalized Estimating Equation		Liner Regression Model		Propensity Score–Adjusted Model	
	Coef/OR (95% CI)	P value	Coef/OR (95% CI)	P value	Coef/OR (95% CI)	P value	Coef/OR (95% CI)	P value
PTH on POD 1^a^	7.10 (1.17–13.03)	0.020*	7.27 (1.42–13.12)	0.015*	5.96 (0.65–11.27)	0.028*	7.29 (1.30–13.28)	0.018*
PTH on POD 30 ^a^	6.26 (−0.69–13.23)	0.077	6.43 (−0.57–12.93)	0.052	4.57 (−1.84–10.98)	0.159	6.49 (−0.52–13.51)	0.069
Serium calcium on POD 1 ^a^	0.05 (0.01–0.09)	0.024*	0.05 (0.02–0.10)	0.016*	0.06 (0.02–0.10)	0.004*	0.05 (0.01–0.10)	0.012*
Hypoparathyroidism^b^			NA		NA			
No	1 (Reference)	NA					1 (Reference)	NA
Yes	0.85 (0.29–2.48)	0.764					0.80 (0.27–2.36)	0.686
Hypocalcemia^b^			NA		NA			
No	1 (Reference)	NA					1 (Reference)	NA
Yes	0.84 (0.25–2.84)	0.780					0.76 (0.22–2.63)	0.663
Identified PGs^b^			NA		NA			
Number ≤ 3	1 (Reference)	NA					1 (Reference)	NA
Number = 4	9.09 (3.29–25.16)	< 0.001*					9.02 (3.25–25.09)	< 0.001*
Preserved PGs in situ^b^			NA		NA			
Number ≤ 3	1 (Reference)	NA					1 (Reference)	NA
Number = 4	5.28 (1.31–21.32)	0.019*					5.12 (1.23–21.26)	0.025*
Autotransplanted PGs^b^			NA		NA			
Number = 0	1 (Reference)	NA					1 (Reference)	NA
Number ≥ 1	2.04 (0.83–5.00)	0.119					2.29 (0.91–5.80)	0.079

ICG, indocyanine green; POD, postoperative day; PGs, parathyroid glands; NA, not available. *Statistically significant.

a The effects of ICGA for dichotomous outcomes were refered to coefficients (β value).

b The effects of ICGA for quralitative outcomes were refered to odd ratios.

### Postoperative PTH and Serum Calcium

As for postoperative PTH, although PTH level remarkably dropped on the first day (**Figure 4B**), PTH and calcium on POD 1 were significantly higher in the ICG group than in the control group (23.16 ± 18.32 vs. 16.06 ± 7.74, P=0.039; 2.13 ± 0.11 vs. 2.08 ± 0.08, P=0.024, respectively) (**Table 2**). While on POD 30, patients in both groups had comparable PTH levels (36.97 ± 17.49 vs. 30.71 ± 13.90, P=0.077) (**Table 2**). Interestingly, postoperative transient hypoparathyroidism and hypocalcemia showed no evident difference between the ICG and control groups (20.59% vs. 23.40%, P=0.764; 14.71% vs. 17.02%, P=0.779, respectively). Additionally, after controlling for confounders in different multivariable models (**Table 3**), the use of ICGA was found to be independently associated with PTH and calcium on POD 1. However, it seemed not to significantly affect PTH on POD 30, transient hypoparathyroidism, and transient hypocalcemia. Additionally, Of the 34 patients in the ICG group, 29 patients had at least one parathyroid gland (ICG score 2), in which three cases developed transient hypoparathyroidism, with recovery on POD 30 for all three patients. In the other five patients without one well-perfused PG, four of these patients developed transient hypoparathyroidism, who also recovered on POD 30. Moreover, As seen in **Figure 4C**, ioPTH 3 and PTH on POD 1 were significantly higher in patients with at least one well-perfused PG than patients without (18.16 ± 13.71 vs. 2.93 ± 2.40, P<0.001; 26.20 ± 18.05 vs. 5.50 ± 5.64, P<0.001, respectively).

### Predictive Ability of ICGA and ioPTH on Transient Hypocalcemia

As indicated in **Table 4**, ioPTH 3 was significantly higher in the patients with hypocalcemia than in the patients without hypocalcemia (5.88 ± 6.03 vs. 17.65 ± 14.06, P=0.008). Additionally, ioPTH 3, relative ioPTH decline, and PTH on POD 1 were significantly associated with postoperative hypocalcemia. The optimal cut-off values for predictors in predicting transient hypocalcemia were ioPTH 3 ≤ 3.37 pg/mL, relative ioPTH decline > 0.838, and PTH on POD 1 ≤ 15.6 pg/mL. The receiver operating characteristic curves for ioPTH 3, and relative ioPTH decline and PTH on POD 1 were shown in **Figure 4D**. Differences between areas under the curve were not significant. A summary of diagnostic characteristics of various predictors (ICG score 2, ioPTH 3, and relative ioPTH decline) was given in **Table 5**. although not significant, the ICG score 2 seemed to be superior to relative ioPTH decline and ioPTH 3.

**Table 4:** Distribution of patients in the ICG group according to predictors of early postoperative hypocalcemia.

Variables	ICG group (n = 34)	Postoperative Hypocalcemia		P-value
		Absent (n = 29)	Present (n = 5)	
**IoPTH level (pg/ml)**				
IoPTH 1^a^	44.51 ± 16.52	45.62 ± 17.60	38.01 ± 4.74	0.349
IoPTH 2^a^	28.98 ± 15.67	30.15 ± 16.67	22.15 ± 3.69	0.030 *
IoPTH 3^a^	15.92 ± 13.79	17.65 ± 14.06	5.88 ± 6.03	0.008 *
ICG score 2 (≥1)				< 0.001*
Present	29	28	1	
Absent	5	1	4	
IoPTH 3^b^				0.029*
> 3.37	28	26	2	
≤ 3.37	6	3	3	
Relative ioPTH decline^b^				0.012*
> 83.8%	9	5	4	
≤ 83.8%	25	24	1	
PTH on POD 1^b^ (pg/ml)				0.005*
> 15.56	21	21	0	
≤ 15.56	13	8	5	

ioPTH, intraoperative measurement of parathyroid hormone; POD, postoperative day. *Statistically significant.

a ioPTH 1, ioPTH 2, and ioPTH 3 are referred to as intraoperative measurement of parathyroid hormone at different time points (after induction, 10 min after removal of one lobe, and 10 min after removal of both lobes, respectively).

b Predictive variables correspond to cutoff values.

**Table 5:** Diagnostic characteristics of different predictors for hypocalcemia.

Measures	Predictors for Hypocalcemia (95% CI)		
	ioPTH decline (≥ 0.838)	ioPTH 3 (< 3.37 pg/ml)	ICG score 2(absent)
Sensitivity	0.80 (0.28–0.99)	0.60 (0.15–0.95)	0.80 (0.28–0.99)
Specificity	0.83 (0.64–0.94)	0.90 (0.73–0.98)	0.97 (0.82–1.00)
Positive predictive value	0.44 (0.14–0.79)	0.50 (0.12–0.88)	0.80 (0.28–0.99)
Negative predictive value	0.96 (0.80–1.00)	0.93 (0.76–0.99)	0.97 (0.82–1.00)
Diagnostic accuracy	0.82 (0.65–0.93)	0.85 (0.69–0.95)	0.94 (0.80–0.99)

ioPTH, intraoperative measurement of parathyroid hormone.

### Complications and Surgical Parameters of IGCA

No ICG-related adverse reactions were observed in the ICG group. Fluorescence was typically observed around 30 seconds to 2 minutes following injection and persisted for approximately 20 minutes. There were no significant differences between the two groups in intraoperative blood loss, postoperative hospital stay time, and transient hoarseness. Notably, the mean surgical time in the ICG group is longer than in the control group (88.82 ± 4.78 vs. 81.28 ± 8.81, P<0.001). The additional cost in the ICG group is only limited to the price of a vial of 25 mg of ICG used per patient (110 Chinese Yuan [equivalent to USD 17])

## Discussion

Postoperative hypoparathyroidism and the resulting hypocalcemia are the most common complications following thyroidectomy (3–6). Due to PGs’ small size, anatomical variation, and similar colors to fat tissue or lymph node, the preservation of PGs and their vascularity remains a challenge even for experienced surgeons, especially when associated with lymph node dissection in cases of thyroid cancer (3, 5, 6). Therefore, Surgical techniques that improve the preservation of the PGs and their blood supply in thyroid cancer surgery are greatly needed. Recently, Intraoperative ICGA has emerged as a promising tool and gained attention in thyroid surgery. It is a simple, rapid and repeatable technique used to identify and preserve PGs, as well as assess the function of PGs and predict postoperative hypocalcemia (8, 9). Contrary to conventional open surgery in many studies, the use of ICGA in robotic surgery omitted an additional laparoscopic imaging system (21). However, there were limited data focused on ICGA applied in thyroid cancer surgery and CND exclusively (12, 21), as well as in robotic surgery (21, 22). Thus, we conducted this study to investigate the utility of ICGA in PTC patients undergoing BABA RT

As for the identification and preservation of PGs, our study suggested that intraoperative ICGA could help identify and preserve more PGs. *Via* ICGA, the PGs and their vascularity could be detected early and identified before the dissection (**Figure 2A**), and therefore be visualized and preserved in real time during the dissection (**Figure 2B**). Thus, ICGA can play an essential role in preventing intraoperative damage to PGs and their vascularization, eventually preserving more PGs in situ, which might also help avoid autotransplantation and accidental removal of PGs. Although the rate of Autotransplanted PGs and Inadvertently resected PGs were almost equivalent between both groups. With more PGs preserved in the ICG group, there was no doubt that postoperative PTH and serum calcium on POD 1 was higher in our study. Whereas postoperative PTH on POD 30 was similar between two groups. Thus, our study indicates that ICGA has a favorable short-term outcome. However, there were no significant differences in transient hypoparathyroidism and hypocalcemia, possibly due to the small sample size and few events. On the other hand, given our postoperative calcium supplementation protocol, the rate of transient hypocalcemia might be low, potentially decreasing our power to detect a significant association of the use of ICGA with transient hypocalcemia. Some studies (16, 21, 22, 28, 29) argued that concomitant fluorescence of the thyroid might hinder parathyroid visual detection, whereas fluorescence of PGs could show an obvious distinction from the fluorescence of thyroid tissue in our study. Several skills might help avoid concomitant fluorescence of thyroid gland in our experience: inferior thyroid artery sealed close to the thyroid tissue before ICG injection if possible; slow injection of ICG; and negative development of thyroid gland and lymph node with carbon nanoparticles. Notably, we have observed that carbon nanoparticles could obviously reduce the fluorescence intensity of thyroid and lymph nodes, with contrast-enhanced fluorescence of PGs. The effect of carbon nanoparticles combined with ICGA on PGs preservation, especially on inferior PGs, warrants further investigation to elucidate the possible mechanism. One challenge in our research is that PGs were identified visually, and no regular biopsy was performed to confirm the presence of parathyroid tissue. While in experienced hands, the rate of correct identification of a structure as a parathyroid should exceed 95%, and it is not the standard of care to biopsy normal PGs. Hence a systematic biopsy is unnecessary (14), much less in robotic thyroidectomy with 15 times magnified three-dimension view. Overall, ICGA is an effective technique in identifying and preserving more PGs, as a result of an improvement of postoperative short-term calcium and PTH levels.

Regarding the evaluation of PGs viability and function, in line with other studies (10, 15, 20), our study suggests that patients with at least one well-perfused PGs (ICG score 2) were closely associated with a higher ioPTH 3 and PTH on POD 1, thereby with a lower rate of transient hypoparathyroidism and subsequent transient hypocalcemia. Some studies (11, 12, 15, 25) reported that patients with at least one well-perfused gland had normal postoperative PTH levels (in the presence of postoperative hypoparathyroidism with a 100% negative predictive value). In contrast, some studies (13, 27, 30) reported fewer positive results regarding the prediction of parathyroid function using ICGA. While in our study, the negative predictive value of patients with at least one well-perfused gland in prediction of postoperative hypoparathyroidism was 89.66% (3 out of 29 patients with at least one well-perfused gland developing transient hypoparathyroidism). Such findings may be attributed to the lack of a standardized objective evaluation of PGs fluorescence. In addition, it is worth noting that one of five patients without one well-perfused gland did not develop transient hypoparathyroidism, suggesting that low-flow ICG patterns are not always corresponding to postoperative hypoparathyroidism, similar to the results of many reports (11, 14, 15, 20, 25, 27), which need further study to elucidate the possible causes and mechanism. Considering that ICGA is capable of the real-time assessment and imaging of PGs perfusion and vascularization, we think ICGA might assist in an immediate decision on autotransplantation. Some authors (13) have reported that ICGA can guide more appropriate autotransplantation without compromising postoperative parathyroid function. However, particular attention should be paid to autotransplantation in special cases where low-flow ICG patterns are not always associated with postoperative PTH change (27). What’s more, the PTH levels on POD 30 were nearly identical not only between the ICG and control group, but also between patients with at least one well-perfused gland and patients without. The results above suggest that partially vascularized (ICG score 1) PGs or/and autotransplanted PGs would recover good parathyroid function in the long term and might be sufficient to avoid long-term hypoparathyroidism.

Regarding ICGA and ioPTH in predicting postoperative hypocalcemia, Our study suggests that ioPTH 3, and relative ioPTH decline could allow an early prediction of postoperative hypocalcemia, with a similar predictive ability to PTH on POD 1, which is in agreement with a recent systematic review (31). In addition, Although not significant, the diagnostic accuracy of ICGA was slightly higher than ioPTH 3 and relative ioPTH decline. There may be a type II error and a larger sample size would find that ICGA could be superior to ioPTH 3 and relative ioPTH decline. The findings above were concordant with recent studies (10, 14, 20). Furthermore, ICGA has advantages over ioPTH in that ICGA allows real-time assessment of individual parathyroid viability and immediate decision-making on autotransplantation without waiting for ioPTH measurement (13, 20). With the accurate prediction of hypocalcemia by ICGA, some studies (11, 15) have demonstrated that the systematic measurement of calcium and PTH level and the systematic supplementation of calcium and vitamin D therapy can be omitted in patients with at least one well vascular gland. Overall, ICGA is an effective method to evaluate PGs viability and function and predict postoperative hypocalcemia, allowing an immediate decision-making on autotransplantation and facilitating postoperative management.

ICGA is a safe technique without any ICG-related adverse reactions in our study. From our experience, the fluorescence of PGs was observed a few seconds after ICG administration (30 seconds to 2 minutes depending on the length of the IV line, the flushing speed, injection speed of ICG, and whether the inferior thyroid artery is sealed before ICG injection). while in other articles (10, 11, 14, 15, 21, 25, 28, 32), PGs were visible from 50 seconds to 3 minutes. As the additional duration of surgery in the ICG group was around 7.5 minutes (around 2.5 minutes per time) with ICGA performed three times during surgery, the mean added time of ICGA is about 5-19 minutes in recent studies (25, 27, 28) during open operation. More time spent in open surgery is required due to additional procedures in preparing a laparoscopic fluorescence imaging system. Regarding costs, as the Firefly system is integrated into the da Vinci robot system, the additional cost is only limited to the price of a vial of 25 mg of ICG used per patient (110 Chinese Yuan [equivalent to USD 17]). Given its potential in possibly omitting the need to measure calcium and PTH levels and supple calcium and vitamin D therapy, even facilitating early hospital discharge, ICGA may be a cost-effective tool in thyroid surgery, especially in robotic surgery.

The present study has several limitations. First, not all 4 PGs were evaluated in some patients. Therefore, in those patients with less than 4 evaluated PGs, the perfusion and viability of the unidentified PGs remained unknown, which might affect the surgery outcome. Secondly, Our research was retrospective in nature, which might have introduced selection biases. Although different models were conducted to control for potential confounders and showed the robustness of the results, a prospective study with large sample size is warranted. Thirdly, there is a lack of consensus on the dose as well as the frequency and timing of ICG administration, and future studies are required to establish standards or formal guidelines that address these questions. Finally, as a qualitative score (ICG 0-2) used in our study was subjective, a quantitative or numerical score of PGs fluorescence by ICGA warrants further investigation.

In conclusion, this study demonstrates that ICGA can allow better identification and preservation of PGs as well as evaluation of PGs viability and function, with the potential for preserving more PGs, guiding more appropriate autotransplantation, and accurately predicting postoperative hypocalcemia. Thus ICGA is a simple, safe, effective and cost-effective tool in PTC patients undergoing BABA RT and CND, capable of improving surgery outcomes and facilitating postoperative management. However, there is no established standard for the use of ICGA, and further research is needed to establish its clinical utility.

## Data Availability Statement

The original contributions presented in the study are included in the article/supplementary material. Further inquiries can be directed to the corresponding authors.

## Ethics Statement

The studies involving human participants were reviewed and approved by Ethics Committee of Xiangya Hospital Central South University. The patients/participants provided their written informed consent to participate in this study.

## Author Contributions

HO, FX, XL: Study design. HO, BS, RC, BW: Collection of data. HO, BW: Analysis and interpretation of data. HO, BS: Writing of manuscript. FX, XL: Decision to submit the article for publication. All authors read and approved the final manuscript.

## Funding

This work was supported by the National Natural Science Foundation of China (grant No. 82073262).

## Conflict of Interest

The authors declare that the research was conducted in the absence of any commercial or financial relationships that could be construed as a potential conflict of interest.

## Publisher’s Note

All claims expressed in this article are solely those of the authors and do not necessarily represent those of their affiliated organizations, or those of the publisher, the editors and the reviewers. Any product that may be evaluated in this article, or claim that may be made by its manufacturer, is not guaranteed or endorsed by the publisher.
